# Chirality-induced bacterial rheotaxis in bulk shear flows

**DOI:** 10.1126/sciadv.abb2012

**Published:** 2020-07-10

**Authors:** Guangyin Jing, Andreas Zöttl, Éric Clément, Anke Lindner

**Affiliations:** 1 School of Physics, Northwest University, Xi’an 710127, China.; 2 Physique et Mécanique des Milieux Hétèrogènes, PMMH, ESPCI Paris, PSL University, CNRS, Sorbonne Université, Université de Paris, 10, rue Vauquelin, 75005 Paris, France.; 3 Institute for Theoretical Physics, TU Wien, Wiedner Hauptstraße 8-10, 1040 Wien, Austria.

## Abstract

Interaction of swimming bacteria with flows controls their ability to explore complex environments, crucial to many societal and environmental challenges and relevant for microfluidic applications such as cell sorting. Combining experimental, numerical, and theoretical analysis, we present a comprehensive study of the transport of motile bacteria in shear flows. Experimentally, we obtain with high accuracy and, for a large range of flow rates, the spatially resolved velocity and orientation distributions. They are in excellent agreement with the simulations of a kinematic model accounting for stochastic and microhydrodynamic properties and, in particular, the flagella chirality. Theoretical analysis reveals the scaling laws behind the average rheotactic velocity at moderate shear rates using a chirality parameter and explains the reorientation dynamics leading to saturation at large shear rates from the marginal stability of a fixed point. Our findings constitute a full understanding of the physical mechanisms and relevant parameters of bacteria bulk rheotaxis.

## Teaser

Experimental orientation distributions reveal scaling laws of bacterial rheotaxis explained using noisy dynamical systems.

## INTRODUCTION

The interaction of swimming microorganisms with flows determines their ability to move in complex environments such as biological channels, soils, or medical conducts. The understanding of the resulting dynamics is crucial for not only a number of societal and environmental challenges, such as infections, soil purification, and contamination of biomedical devices but also cell sorting and analysis (*1*–*4*). Particularly interesting are situations where microorganisms do not just follow the local flow velocity to be transported downstream along stream lines but orient with respect to the flow and show preferential transport up- or downstream or even a sidewise drift. This nontrivial organization under flow is expected to be at the origin of transport anomalies observed in the dispersion process in capillary tubes or porous media, which remains poorly understood today (*5*–*7*). From a fundamental point of view, these transport dynamics are determined by the microorganism shape, activity, and rotational (or run-and-tumble) noise in combination with the given flow properties.

Passive nonchiral rigid particles transported in viscous flows generally follow streamlines, even if complex orientation dynamics can be present as a function of the particle shape (*8*). For example, elongated passive objects in shear flows perform so-called Jeffery orbits (*8*, *9*), periodically changing their orientation while being transported downstream along stream lines. These orbits have been observed in different experimental systems with and without Brownian noise (*10*–*12*), and the role of fluctuations on the orbits has been addressed theoretically (*12*).

For passive particles, drifts along velocity gradients have only been observed in more complex situations and in the presence of shear gradients, for example, in viscoelastic flows (*13*), in the presence of inertia (*14*), or for flexible particles (*15*, *16*). When particle symmetry is broken by chirality, particles can migrate toward the vorticity directions, i.e., perpendicular to velocity gradients, as has been predicted and experimentally observed (*17*, *18*). Whether a drift toward the right/left (positive/negative vorticity direction) is observed depends on the handedness of the particle and the sign of the local shear rate. For all these systems, drift velocities remain small compared to flow velocities and their influence only becomes noticeable after long distances.

This changes fundamentally when particles become active. For motile microorganisms, orientation dynamics, mainly governed by Jeffery dynamics (*9*), directly translate into swimming directions and drift velocities become of the order of swimming velocities. In shear flow, microswimmers crossing streamlines lead to new families of “active Jeffery orbits.” In Poiseuille flow, “swinging” and “shear-tumbling” trajectories (*19*, *20*) were identified theoretically and numerically, and their existence was recently confirmed experimentally for motile *Escherichia coli* bacteria (*21*). Moreover, in Poiseuille flow, kinetic theory (*22*) predicts that the interplay between stochastic reorientation, active swimming, and the varying local shear rate leads to preferred upstream and downstream swimming.

Microorganism transport has primarily been studied close to solid surfaces, mainly due to the fact that activity leads to surface accumulation (*23*). In addition, in these regions, transport velocities are small, while large velocity gradients, in combination with specific surface interactions, strongly influence microswimmer dynamics. It has been found half a century ago (*24*) that microorganisms can orient with respect to flow gradients to surfaces. Upstream orientation has been observed for sperm cells (*24*–*26*), for *E. coli* bacteria (*27*–*30*), and artificial microswimmers (*31*–*34*). This upstream motion has been analyzed theoretically (*33*–*36*) and is generally attributed to fore-aft asymmetry of the swimmer shape. In addition, organisms reorient, on average, toward the positive vorticity direction (*27*–*29*, *37*), an effect attributed to the counterrotation of cell body and flagella. At high enough shear rates, the interplay of different effects can lead to oscillatory surface rheotaxis (*38*).

In the bulk, bacterial rheotaxis has been found by Marcos *et al.* (*37*) who have shown that surface interactions are not required to observe bacteria drift toward the vorticity direction but that a helical flagella shape leading to chirality-induced lift forces was sufficient. In combination with the viscous drag on the bacteria head, this leads to a rheotactic torque (*37*) reorienting bacteria in flow resulting in a net rheotactic drift, which has the opposite sign compared to the drift of passive helices (*18*) and has been experimentally observed for swimming *Bacillus subtilis* at a specific fixed distance away from the walls of a microchannel Poiseuille flow (*37*).

The rheotactic drift has been characterized in the form of a mean rheotactic velocity as a function of the local shear rate and was quantitatively reproduced using a hydrodynamic model of a microswimmer based on resistive force theory in combination with Brownian noise (*37*). Despite the fact that the numerical model presented by Marcos *et al.* (*37*) reproduces quantitatively, by parametric adjustment, the mean rheotactic drift velocity, the details of the underlying physical mechanisms were not fully revealed. Moreover, the absence of an analytical model did not allow retrieving the scaling laws of the dependence of the mean rheotactic velocity on the shear rate. In addition, no interpretation of the effect in relation with the relevant physical parameters could be established. Last, no experimental data on the distribution of bacteria orientations were given by Marcos *et al.* (*37*), which we show is crucial to understand the observed mean rheotactic velocities.

To better understand the rheotactic phenomenology for swimming bacteria in shear flows, we combine an experimental, numerical, and theoretical analysis. Microfluidic experiments are performed using wild-type *E. coli* bacteria in wide channels, and a large number of bacteria tracks are recorded as projections into planes parallel to the bottom wall at different distances from the latter. Careful tracking of active bacteria, as well as passive tracers, allows them to obtain precise rheotactic velocity and orientation distributions at different positions of the Poiseuille flow at various flow rates. To quantitatively match the experimental results, we propose an extended analytical description of the swimming kinematics reflecting the chiral nature of the propelling flagella in addition to the standard Jeffery dynamics and rotational and run-and-tumble noise. The combined role of the drag on the bacterial body and the flagellar chiral structure are encoded into a single number, the chiral strength, reflecting the reorientation effect in response to the local velocity gradients. An empirical value of this coupling parameter is determined for a wild-type *E. coli*.

Theoretical analysis reveals the scaling laws behind the average rheotactic velocities for a full range or shear rates. We define a dimensionless shear rate in the form of a chirality number combining the effects of chiral strength and rotational Péclet number, leading to a collapse of all experimental and numerical datasets obtained from a parameter study. At low and moderate shear rates, we reveal that the rheotactic drift velocity increases linearly with the chirality number. At larger chirality number, from a linear stability analysis around a marginally stable fixed point, we explain the reorientation dynamics leading to a very slow approach of the rheotactic drift toward the swimming velocity value. Our findings constitute a full characterization of bacteria rheotaxis under shear flow, spanning the complete space of the Poiseuille channel flow and a comprehensive understanding of the physical mechanisms and relevant parameters behind it.

## RESULTS AND DISCUSSION

### Mean rheotactic velocity

**Fig. 1:**
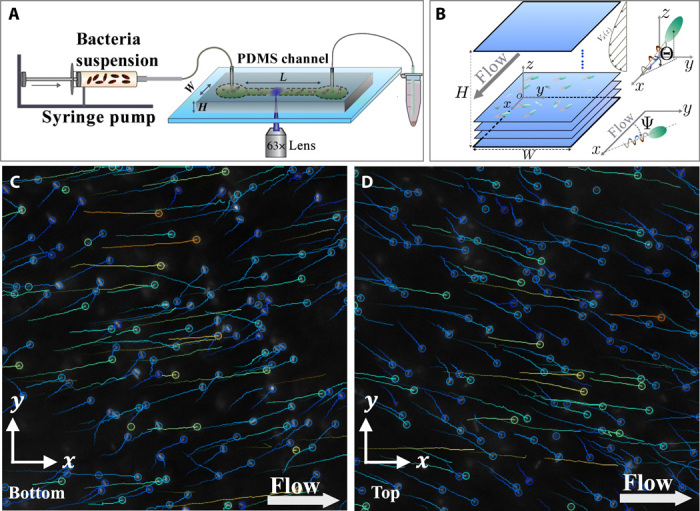
Setup and typical trajectories of swimming bacteria in the upper and lower half of the channel. (**A**) A dilute bacterial suspension is injected into a polydimethylsiloxane (PDMS) microchannel (width *W* = 600 μm, height *H* = 100 μm, and up to *L* = 20 mm in length) at a given flow rate *Q*. (**B**) Bacteria and passive tracers are recorded at 200 frames per second (fps) using a 63× lens (observation window in the *x*-*y* plane, 200 μm by 100 μm) at varying distances *z* from the bottom wall. The angle Ψ defines the bacteria orientation in the *x*-*y* plane, and Θ is the out-of-plane angle. (**C** and **D**) Typical trajectories of swimming bacteria in the lower (C) and upper channel half (D). The circles represent the end of the bacteria trajectories. Bacteria drift toward the right with respect to the negative flow direction in (C) and in the opposite direction in (D).

A dilute suspension of *E. coli* bacteria is injected at a given flow rate *Q* into a microchannel of width *W* = 600 μm and height *H* = 100 μm (Fig. 1A), which imposes to a very good approximation a planar Poiseuille flow *V_x_*(*z*) = 4*V*_max_*z*(*H* − *z*)/*H*^2^ sufficiently away from the side lateral walls, with *V*_max_, the flow velocity in the center of the channel. The local shear rate is then $\dot{\gamma }=\partial V_{x}(z)/\partial z=4V_{\text{max}}(H-2z)/H^{2}$. Using a high-magnification objective lens, bacteria tracks are recorded as projections into the *x-y* plane at different distances from the bottom wall (Fig. 1B) and far away (≳250 μm) from lateral side walls. Exact calibration of the local flow velocities and channel orientation is obtained by the simultaneous recording of passive tracers for all experiments performed. This calibration step is essential for the determination of bacteria orientation and velocity distributions. Since we focus on bulk dynamics, bacterial trajectories close to the top and bottom channel walls are not included in our analysis.

Figure 1 (C and D) show examples of bacterial trajectories in layers in the upper and lower parts of the microchannel Poiseuille flow. One clearly sees a bias toward the right with respect to the negative flow direction on (C) and toward the left on (D), confirming the rheotactic cross-streamline migration in the *y* direction induced by the chirality of the left-handed bacteria flagella (*37*) and being a function of the sign of the local shear rate.

**Fig. 2:**
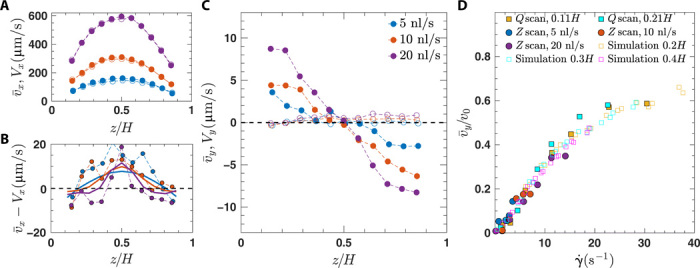
Mean velocities for bacteria and passive tracer beads. (**A**) Bacteria [${\bar{v}}_{x}(z)$] and bead [*V_x_*(*z*)] velocities obtained by scanning through the *z* direction. Passive tracer beads with diameter *d* = 1 μm (empty circles) and bacteria (filled circles) are represented at mean flow rates of *Q* = 5 (blue), 10 (red), and 20 nl/s (purple). (**B**) Difference between bacteria and flow velocities ${\bar{v}}_{x}-V_{x}$. Results from simulations are shown by solid lines. (**C**) Corresponding mean rheotactic velocities ${\bar{v}}_{y}$ and bead velocities *V_y_*(*z*). (**D**) Rheotactic velocity ${\bar{v}}_{y}$ normalized by the average bacteria swimming speed *v*_0_ as a function of local shear rate $\dot{\gamma }$ controlled by two methods: *z* scan, scanning through the channel height at given flow rates *Q* (5,10, and 20 nl/s), corresponding to the data of (B), and *Q* scan, varying flow rates at fixed channel height (0.11*H* and 0.21*H*). Results from simulations are indicated by open symbols and are given for heights 0.2*H*, 0.3*H*, and 0.4*H*.

Quantitative measurements of mean bacteria velocities (${\bar{v}}_{x}$,${\bar{v}}_{y}$) and transport velocities of passive tracers (*V_x_*,*V_y_*) for different flow rates *Q* are displayed in Fig. 2 (A and C) in the flow direction (*x* direction) and in the vorticity direction (*y* direction), respectively, as a function of the distance *z* from the bottom wall. The passive tracers (open symbols) follow a Poiseuille profile [*V_x_*(*z*) in Fig. 2A] without a drift in the *y* direction (Fig. 2C), indicating the perfect alignment of channel and microscope, and the *x* axis is identical to the flow direction.

Different local shear rates $\dot{\gamma }$ can be obtained in two ways, either by varying *V*_max_ via the imposed flow rate *Q* or by varying the position *z* inside the channel. For a systematic analysis of bacterial rheotaxis, allowing to independently investigate the role of local shear rate and positions within the Poiseuille flow, both *Q* scans and *z* scans are performed.

Mean bacteria velocities (filled symbols) deviate from the background velocities both in the parallel (*x*) direction and in the transverse (*y*) direction. In the *x* direction, bacteria velocities are higher in the center of the channel and lower close to channel boundaries compared to the background flow. This is illustrated more clearly in Fig. 2B where the *z*-dependent relative velocities between bacteria and background flow are shown. This indicates a preferred orientation of bacteria downstream in the channel center and upstream closer to the channel walls for all considered flow rates. Mean bacteria velocities ${\bar{v}}_{y}$ confirm the visual observations from Fig. 1 (C and D) and have opposite signs in the lower and upper half of the Poiseuille flow. This rheotactic velocity ${\bar{v}}_{y}$ increases with increasing flow rate *Q* and with decreasing distance *z* from the wall. Note that wall effects are visible for the data points at a distance of 0.1*H* (∼10 μm) from both top and bottom walls and will be excluded from further analysis in this paper.

Figure 2C shows mean drift velocities ${\bar{v}}_{y}$ as a function of local shear rates from different datasets, including the results from the three *z* scans from Fig. 2B and from two different *Q* scans. We note that we scale the velocities by the average bacteria velocity *v*_0_ = 25 μm s^−1^ in the absence of flow (see the Supplementary Materials). Representing the mean velocities as a function of the local shear rate leads to a reasonable data collapse, indicating that the local shear rate is the main control parameter of our system. Within our range of shear rates, the increase in mean drift velocity is first linear with local shear rate, similar to what was observed in (*37*), and then reaches a maximum of around half the average bacteria swimming velocity at the highest shear rate.

### Theoretical framework

To understand the physical mechanisms behind bacterial rheotaxis in microchannel flow, we develop a theoretical framework that captures the dynamics of individual noninteracting bacteria. We account for the elongated and chiral shape of the flagellated bacteria, their self-propulsion velocity *v*, tumbling, translational and rotational noise, and their advection and rotation in Poiseuille flow. Denoting the instantaneous position of a bacterium by **r** and its orientation by **e**, we can write the dynamics of a bacterium in Poiseuille flow as

$$\frac{dr}{\mathrm{dt}}=ve+V_{x}(z)+ℋ\cdot \xi$$ (1)

for the positional dynamics and

$$\frac{de}{\mathrm{dt}}=[\Omega^{J}+\Omega^{C}+\sqrt{2D_{r}}\xi^{r}]\times e$$ (2)

for the orientational dynamics. For Poiseuille flow, these two equations are coupled, and Eq. 1 includes orientation-dependent self-propulsion, advection in Poiseuille flow, and anisotropic translational diffusion, and Eq. 2 includes position-dependent reorientation in flow. The random numbers ξ*_i_* and $\xi_{i}^{r}$ model Gaussian white noise (see Materials and Methods); ℋ is related to the anisotropic translational diffusion tensor (see Materials and Methods), and *D_r_* is the rotational diffusion constant. We use the well-known Jeffery reorientation rate **Ω***^J^* to capture the rotation in flow due to elongation (*9*, *20*, *21*, *39*)

$$\begin{array}{c} \Omega_{x}^{J}=\dot{\gamma }\frac{1}{2}Ge_{x}e_{y}\\ \Omega_{y}^{J}=\dot{\gamma }\frac{1}{2}(1+G(e_{z}^{2}-e_{x}^{2}))\\ \Omega_{z}^{J}=-\dot{\gamma }\frac{1}{2}Ge_{y}e_{z} \end{array}$$ (3)

with the Bretherton shape factor *G* = (α^2^ − 1)/(α^2^ + 1) ≲ 1, where α is the effective aspect ratio of the bacteria. The chiral strength ν of a bacterium is related to the specific shape of the left-handed chiral flagella bundle and the size of the head (*37*, *38*), leading to a chirality-induced reorientation rate **Ω***^C^*, as derived in (*38*)

$$\begin{array}{c} \Omega_{x}^{C}=-\nu \overset{\cdot }{\gamma }e_{z}(2e_{x}^{2}-1)\\ \Omega_{y}^{C}=-2\nu \overset{\cdot }{\gamma }e_{x}e_{y}e_{z}\\ \Omega_{z}^{C}=-\nu \overset{\cdot }{\gamma }e_{x}(2e_{z}^{2}-1) \end{array}$$ (4)

The same result has been rederived recently in (*40*). To model tumbling, we include tumble events that modify the bacterium orientation instantaneously at exponentially distributed times (see Materials and Methods). We note that we neglect a term in Eq. 1 that captures a small passive rheotactic drift force but is negligible compared to the other terms for our regime of parameters.

The theoretical model (Eqs. 1 to 4) contains a number of parameters that need to be determined. The swimming speed *v* of individual bacteria is assumed to be normally distributed with mean *v*_0_ = 25 μm s^−1^ and SD of 8 μm s^−1^ to closely match experimental conditions in the absence of flow (see the Supplementary Materials). While the value of *D_r_* = 0.057 s^−1^ (*41*) and the tumbling statistics (*42*) can be estimated from the literature, we treat α and ν as free parameters. These parameters can be adjusted using the precise experimental results. We thus perform Brownian dynamics simulations of the coupled Eqs. 1 and 2 including tumbling and simple steric repulsion for swimmer-wall interactions to be compared to the experimental results for the mean rheotactic velocities ${\bar{v}}_{y}$ as a function of the local shear rate $\dot{\gamma }$ as shown in Fig. 2C.

The initial slope of ${\bar{v}}_{y}(\dot{\gamma })$ can be used to adjust the mean chiral strength of the bacteria to ν = 0.06, being of the same order as estimated previously using resistive force theory (*37*, *38*). The parameter ν only depends on the bacterial shape. In particular, its value depends nontrivially on the helical shape of the flagella bundle, is maximized for large cell bodies, and vanishes in the limit of very small cell body size. The effective aspect ratio α is determined from the deviation from the linear regime toward the saturation of the rheotactic velocity at high shear rates, with the results not being very sensitive to the value of the parameter α. Here, we use α = 5, in agreement with typical experimental values (*21*). These values for α and ν are used for all further comparison between experiments and simulations.

From Fig. 2D, we can see that by adjusting these parameters, the numerical model describes very well the experimental observations. As in the experiments, data obtained from different positions *z* in the channel and different flow strength collapse onto almost a single curve, but small deviations stem from the velocities obtained from different layers in the channel.

In Fig. 2B, we compare the longitudinal velocity differences between bacteria and the Poiseuille flow from numerical simulations with the experiments, which show the same results, i.e., a preferred upstream orientation near the walls and a downstream orientation in the center. This is in excellent agreement with a kinematic model, which neglects the swimmer chirality (*22*). This indicates that chirality does not influence, at least qualitatively, the polarization profile along the flow direction but mainly affects the bacteria orientations in the transverse direction.

### Velocity and orientation distributions

**Fig. 3:**
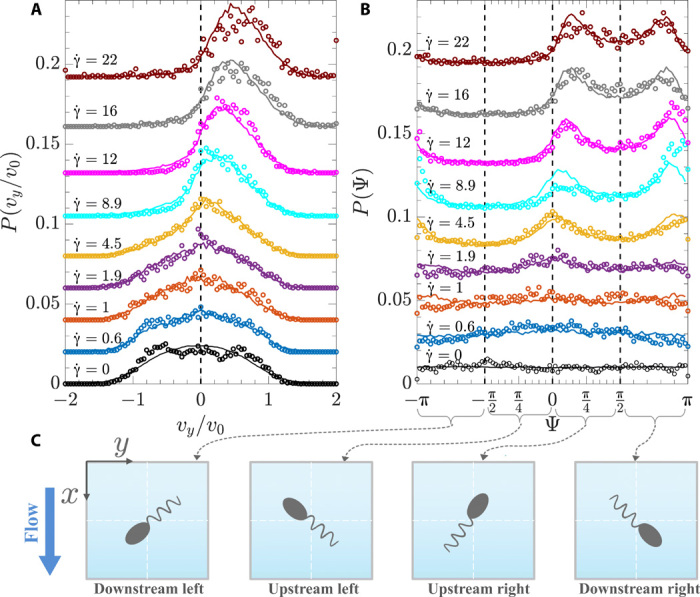
Velocity and orientation distributions. Experimental (symbols) and numerical (solid lines) results of (**A**) velocity *v_y_* and (**B**) orientation Ψ distributions obtained by varying the flow rate *Q* at a given distance from the channel bottom wall (*z* ≈ *H*/4). Local shear rates have been closely matched between experiments and simulations. For better readability, the different curves are shifted in the vertical direction. (**C**) Sketch of the bacterium orientation Ψ and relation to up- and downstream and left and right orientation.

We now turn to velocity and orientation distributions. Figure 3A shows the rheotactic velocity distributions *P*(*v_y_*) at a given height *z* = *H*/4 for different local shear rates corresponding to the mean values shown on Fig. 2C. As expected, without flow and for small shear rates, the velocity distribution is very broad and centered around zero. For increasing shear rates, the distributions are shifted more and more toward positive *v_y_* values, together with a small decrease of the width of the distribution. The experimental and numerical distributions are in excellent agreement and reinforce the validity of our model. Note that using a swimming velocity distribution matching the experimental results is crucial to obtain such an agreement, whereas it is sufficient to work with average values for α and ν.

The rheotactic velocity of each bacterium is a direct consequence of its three-dimensional (3D) orientation and its intrinsic swimming velocity *v* setting the instantaneous velocity *v_y_*(*t*) = *ve_y_* = *v* cos Θ(*t*) sin Ψ(*t*). Here, the angle Θ is linked to the orientation of the bacterium in the *z* direction, *e_z_* = sin Θ, and *e_x_* = − cos Θcos Ψ, and *e_y_* = cos Θsin Ψ (see Fig. 1B). While bacteria orientations can directly be determined from the numerical simulations, only projections of bacteria trajectories into the *x-y* plane are accessible from the experiments and the in-plane orientations are obtained from the orientation of the velocity vector (see Materials and Methods), defined by the angle Ψ. We thus show the corresponding orientation distributions *P*(Ψ) for different local shear rates in Fig. 3B for the layer *z* = *H*/4. Figure 3C shows a sketch of the bacterium orientation Ψ and relation to up- and downstream and left and right orientation. At zero shear rate, the orientation distribution is flat and remains so for small shear rates, indicating no preferred bacteria alignment under weak flow. With increasing shear rate, bacteria orient, on average, more and more toward positive values of Ψ and thus toward the right with respect to the negative flow direction. In addition, a double peak is emerging, which is found to be mostly symmetric around Ψ = π/2, corresponding to a bacteria orientation perpendicular to the flow direction. Bacteria are thus oriented up and downstream around the perpendicular orientation. These peaks become more pronounced and also move closer together with increasing shear rate. They correspond to a single peak in the velocity distributions as seen on Fig. 3A.

**Fig. 4:**
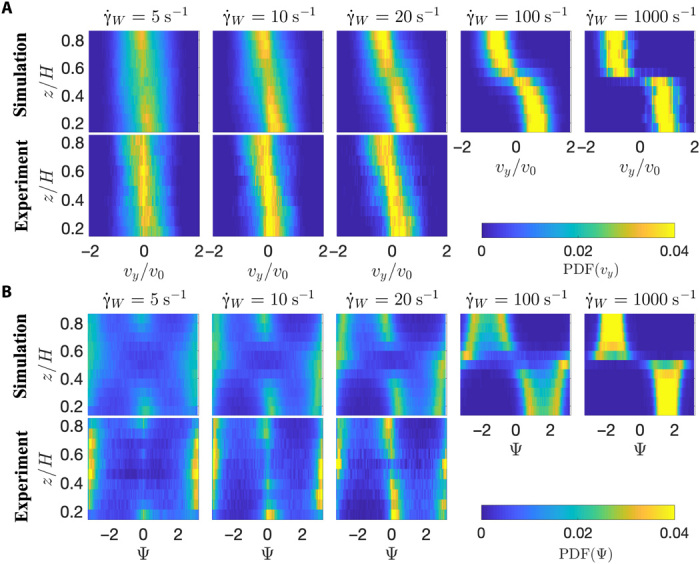
Color map of rheotactic velocity and orientation distributions as a function of channel height from experiments and simulations. (**A**) Probability density function (PDF) for the rheotacitic velocity *v_y_*. (**B**) PDF for the swimmer orientation Ψ. Different panels correspond to different applied flow rates, as indicated by the corresponding wall shear rates ${\dot{\gamma }}_{w}$.

To represent a more complete picture of the orientation distributions under flow, Fig. 4 shows the distributions layer by layer along the channel height. *z* scans have been performed at different imposed flow rates *Q* and are represented as a function of the wall shear rate ${\dot{\gamma }}_{W}=4V_{\text{max}}/H$. Higher flow rates are displayed only for the simulation results, as at those large flow velocities, the time resolution of the experimental image capture is not sufficient to resolve the bacteria trajectories. The probabilities of the rheotactic velocity and orientation distributions are represented with a color code where yellow corresponds to a high probability and blue corresponds to low probability. Peaks in the distributions are thus easily identified as regions of yellow color. For low flow rates, the continuous shift of the peak velocity from zero toward higher velocities when moving away from the middle of the channel toward the channel walls (and thus with increasing local shear rate) is clearly visible, in agreement with Fig. 2C. For larger flow rates, in layers closer to the channel walls and thus for higher local shear rates, only a very weak increase or even a saturation of this peak value can be identified, in particular, from the numerical results. From the orientation distributions, the existence of a double peak is clearly visible for moderate flow rates and a decrease of the distance between the two peaks is observed when getting closer to the channel walls. At high flow rates, the simulation results show that the double peak merges into a single but wide peak.

These figures also show the up- and downstream orientations induced by the swimming dynamics of the bacteria in the Poiseuille flow, where bacteria cross multiple layers in the *z* direction during a trajectory (*19*, *21*, *22*, *43*). This is, in particular, visible at small wall shear rates, where in the center of the channel, most bacteria are oriented downstream, as can be seen by the yellow peak close to π. Closer to the channel walls, this peak is found around 0, corresponding to an upstream orientation, in agreement with the relative bacteria velocities shown in Fig. 2B.

### Theoretical understanding of the observed orientation distributions

To explain the orientation distributions *P*(Ψ, Θ) at the origin of the rheotactic behavior, we start by discussing a simple model system for bulk rheotaxis, namely nontumbling elongated bacteria in linear shear flow with constant shear rate $\dot{\gamma }$. In the absence of noise, the equations for the orientations of chiral microswimmers (Eq. 2) can be rewritten in terms of the angles Ψ and Θ and read [see also (*38*)]

$$\begin{array}{c} \dot{\Psi }=\frac{\dot{\gamma }}{2}(1+G)\text{sin}\;\Psi \;\text{tan}\;\Theta +\dot{\gamma }\nu \;\text{cos}\;\Psi \frac{\text{cos}\;2\Theta }{\text{cos}\;\Theta }\\ \dot{\Theta }=\frac{\dot{\gamma }}{2}(1-G\;\text{cos}\;2\;\Theta )\text{cos}\;\Psi +\dot{\gamma }\nu \;\text{sin}\;\Psi \;\text{sin}\;\Theta \end{array}$$ (5)

Both the Jeffery (first terms) and the rheotactic contributions (second terms) are linear in the shear rate $\dot{\gamma }$, which, as a consequence, only determines the time scale of the dynamics.

**Fig. 5:**
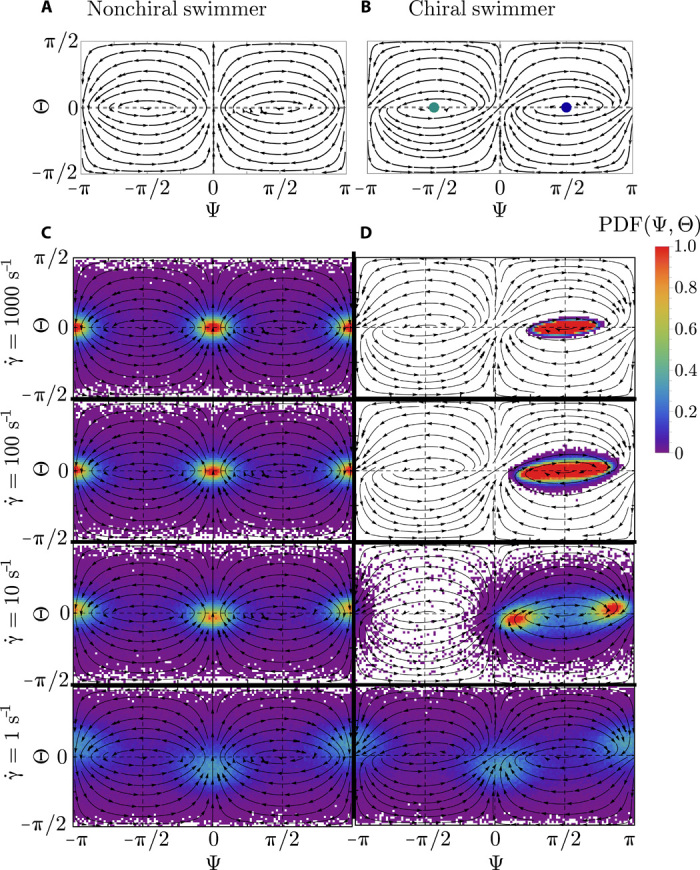
Orientation phase space and simulated probability distributions for nontumbling bacteria in simple shear flow. (**A**) The streamlines of a nonchiral swimmer simply follow Jeffery’s periodic solutions for passive ellipsoids. (**B**) Chirality breaks the left-right symmetry, which is the main reason for bacterial bulk rheotaxis. The green and blue dots correspond to marginally stable fixed points in the linear regime pointing to the left (Ψ = − π/2, Θ = 0) and to the right (Ψ = π/2, Θ = 0) side of the channel. (**C** and **D**) Orientation distributions for nonchiral (C) and chiral (D) swimmers at different shear rates using our standard parameters (ν = 0.06, *D_r_* = 0.057 s^−1^, α = 5).

The orientation (Ψ(*t*), Θ(*t*)) of nonchiral swimmers (ν = 0) simply follow Jeffery orbits, which are solutions of Jeffery’s equations of motion (see also Eq. 3). The Jeffery orientation phase space is shown in Fig. 5A, and closed streamlines indicate the well-known periodic Jeffery solutions that depend on the initial orientation of the swimmer and correspond to a constant of motion, the so-called Jeffery constant (*9*). The arrows indicate the direction of motion on the unit sphere. Again, the shear rate $\dot{\gamma }$ does not alter the Jeffery orbits but only sets the time scale of how fast an elongated particle or bacterium rotates. Note that when a particle starts oriented to the left/right (Ψ < 0/Ψ > 0), it never switches to the other side.

Adding the rheotactic term now breaks the left-right symmetry, and as can be seen in Fig. 5B, all orientations eventually end up on the right (Ψ > 0), leading to the mean rheotactic velocity ${\bar{v}}_{y}>0$. To understand the orientational behavior in more detail, we see that (Ψ* = ± π/2, Θ* = 0) are two fixed points of the system (Eq. 5), defined where $\left({\dot{\Psi }}^{*},{\dot{\Theta }}^{*})=(0,0\right)$. The eigenvalues of the stability matrix for the linearized system around the fixed points can be evaluated to

$$\lambda_{1,2}=\pm \frac{i}{2}\dot{\gamma }\sqrt{1-G^{2}-4\nu^{2}}=\pm i\omega$$ (6)

These Eigenvalues have a vanishing real part, which means that the fixed points are actually not stable/unstable but marginally stable. In other words, the typical time scale at which a bacterium orients toward the fixed point on the right diverges, and it will therefore never be reached. We observe that simulated trajectories without noise do not reach the fixed point but get trapped in periodic orbits around the marginally stable fixed point.

We now turn to the question of how rotational noise, always present in experiments, influences the orientational distribution of bacteria in flow. The rotational diffusion constant *D_r_* of the bacterium compared to the strength of the shear rate $\dot{\gamma }$ now plays an important role. For nonchiral swimmers (ν = 0), noise affects the dynamics similar to passive elongated particles (*44*). Because of noise, trajectories now erratically move around between different Jeffery orbits (*12*), which strongly influences the orientation distribution function *P*(Ψ, Θ), as shown in Fig. 5C. Here, we show results of 1000 averaged steady-state trajectories started uniformly distributed on the unit sphere for a fixed rotational diffusion *D_r_* = 0.057*s*^−1^ but for a broad range of different shear rates. Purple and blue correspond to low probability, while yellow and red correspond to high probability (see scale bar). The first row in Fig. 5C shows the case where noise is not yet important because of the high shear rate $\dot{\gamma }=1000s^{-1}$, and *P*(Ψ, Θ) almost follows the deterministic solution obtained from Eq. 5. The peaks at (Ψ = 0, Θ = 0) and (Ψ = π, Θ = 0) correspond to particles aligned with the flow, upstream or downstream, respectively. These peaks are a consequence of the fact that elongated particles moving on a (typically kayaking) Jeffery orbit have a high probability to come close to these positions, which can be seen by the increased density of streamlines in Fig. 5A. Together with a decreased rotation rate near these points, this leads to the large probability observed. Note that the slowest rotation is near the log-rolling states (Ψ = ± π/2), but hardly any initial condition leads to orbits that come close to these states and precludes high probabilities around them.

As can be seen in the second row in Fig. 5C, reducing the shear rate by a factor of 10 does not change the distributions substantially, meaning that noise is still not important for nonchiral swimmers. This changes for smaller shear rates, $\dot{\gamma }=10\;s^{-1}$, where the peaks of the distributions become less pronounced. At even smaller flow rates, $\dot{\gamma }=1\;s^{-1}$ (last row), noise randomizes the orientations almost completely, and only very weak peaks remain. Note that both upstream and downstream peaks are not symmetrically distributed around Θ = 0 for small $\dot{\gamma }$ because of a subtle interplay between noise and flow, which leads to nonuniform microswimmer profiles in the *z* direction (*22*, *43*, *44*). In general, the important physical quantity that determines the nonchiral orientation distribution is the rotational Péclet number ${\text{Pe}}_{r}=\dot{\gamma }/D_{r}$. As we will see in the following, the situation is less simple when particles are chiral.

For chiral microswimmers (ν > 0), the signatures of the marginally stable fixed point can be seen at high shear rates. As shown in the first row of Fig. 5D, even for very weak noise or high shear rates ($\dot{\gamma }=1000s^{-1}$) the peak of the distribution is not a simple δ peak but smeared out around the marginally stable fixed point at Ψ = π/2. For smaller shear rate $\dot{\gamma }$, when noise becomes more dominant, the probability distribution becomes even broader and develops bimodal peaks, which shift more and more toward orientations aligned (or antialigned) with the flow when lowering $\dot{\gamma }$ further (second and third row in Fig. 5D). The positions of these peaks are triggered by a competition between the chirality-induced attraction to the right and the Jeffery peaks, which become more and more dominant for smaller shear rates, since noise then helps to move around in the phase space more easily. For low $\dot{\gamma }$ (last row), chiral migration to the right is very inefficient compared to noise, and the probability distributions look very similar to the nonchiral system.

Last, we note that the frequency of oscillation ω (see Eq. 6) is comparable to the pure Jeffery frequency $\omega_{J}=\dot{\gamma }\sqrt{1-G^{2}}/2$ but is reduced by approximately 5% for our standard parameters ν = 0.06 and α = 5.

### Universal scaling and master curve

**Fig. 6:**
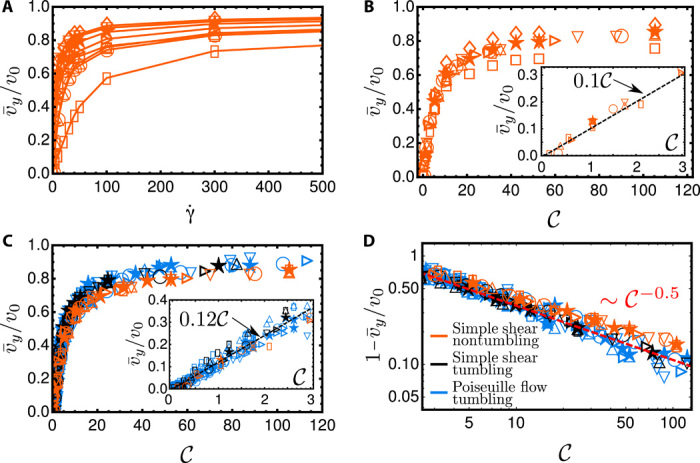
Universal scaling of the rheotactic velocity. (**A**) Dependence of the scaled mean rheotactic velocity *v_y_*/*v*_0_ on the shear rate $\dot{\gamma }$ for nontumbling bacteria in simple shear (simulations) for different parameter sets (rotational diffusion *D_r_*, bacterium aspect ratio α, chiral strength ν). (**B**) Results as shown in (A) but plotted against the chirality number 𝒞. (**C**) Data in (B) for α = 5 compared to tumbling bacteria in simple shear flow and Poiseuille flow. (**D**) Slow algebraic saturation at high shear rates. Color code indicated in (D). Symbol code used in all subfigures: ⋆*D_r_* = 0.057, α = 5, ν = 0.06; ▯*D_r_* = 0.057, α = 5, ν = 0.006; ○*D_r_* = 0.2, α = 5, ν = 0.06; △*D_r_* = 0.057, α = 5, ν = 0.02; ▽*D_r_* = 0.057, α = 5, ν = 0.1; ⊳*D_r_* = 0.1, α = 5, ν = 0.06; ◊*D_r_* = 0.057, α = 3, ν = 0.06; □*D_r_* = 0.057, α = 10, ν = 0.06.

We now analyze the dependence of the rheotactic drift on the system parameters. Therefore, we simulate the dynamics of different rotational diffusion *D_r_*, bacterium aspect ratio α, and chiral strength ν for varying shear rates $\dot{\gamma }$. Figure 6A shows the scaled mean rheotactic velocities ${\bar{v}}_{y}/v_{0}$ as a function of the shear rate for different nontumbling bacteria in simple shear flow. As expected, none of the curves approach ${\bar{v}}_{y}/v_{0}\to 1$ even for very large shear rates because of the aforementioned trapping and oscillations of the swimmer orientations in the vicinity of the marginally stable fixed point (Ψ* = π/2, Θ* = 0).

As seen before, for small shear rates, all curves increase linearly with the shear rate (Fig. 2D). The reason is that for small shear rates, the peaks of the distributions are close to Ψ^±^ = {0, π} and Θ^±^ = 0 (see also Fig. 3B), and the peaks linearly shift away from them with increasing shear rate. The slope is stronger for larger chirality ν or weaker rotational Brownian noise *D_r_*.

To understand the behavior at small shear rates, we linearize Eq. 5 around (Ψ^±^, Θ^±^), i.e., evaluate it at angles Ψ = Ψ^±^ + ϵ_Ψ_ and Θ = Θ^±^ + ϵ_Θ_ with small ϵ_Ψ_ and ϵ_Θ_. To the lowest order in ϵ_Ψ_ and ϵ_Θ_, we then obtain $\dot{\Psi }(\Psi ,\Theta )=\pm \dot{\gamma }\nu$ + h.o.t. and $\dot{\Theta }(\Psi ,\Theta )=\Omega_{\Theta }^{J}$ + h.o.t., where all higher-order terms (“h.o.t.”) are at least quadratic in the angles ϵ_Ψ_ and ϵ_Θ_ and $\Omega_{\Theta }^{J}$ is the Jeffery contribution to the angular velocity component Ω_Θ_. The reorientation rate $\dot{\Psi }$, which is responsible for the drift toward the right, is to the lowest order independent of *G* and linear both in $\dot{\gamma }$ and in ν. This suggests that the shifts of the peaks Ψ* of the probability distributions, and, hence, of the mean values ${\bar{v}}_{y}$, should be a function of $\dot{\gamma }\nu$, acting against the noise quantified by *D_r_*.

Our analysis and the results shown in Fig. 6A now suggest introducing a dimensionless chirality number

$$C=\frac{\overset{\cdot }{\gamma }\nu }{D_{r}^{\text{eff}}}$$ (7)

as the main relevant physical quantity, which regulates rheotaxis. Here, we use $D_{r}^{\text{eff}}$ as the effective rotational diffusion constant determined via the exponential decay of the bacterium orientation autocorrelation function in the absence of flow, $\langle e(0)\cdot e(t)\rangle =e^{-2D_{r}^{\text{eff}}t}$ (*23*). It is simply the rotational diffusivity *D_r_* for nontumbling bacteria but larger for tumbling bacteria because of the extra tumbling reorientations and estimated to $D_{r}^{\text{eff}}\approx 0.25\;s^{-1}$ in our experiments. Note that together with the experimentally explored range of local shear rates $\dot{\gamma }\approx 0\;\text{to}\;50\;s^{-1}$, this corresponds to a range of Péclet number Pe ≈ 0 to 200.

In Fig. 6B, we now show the same data as in Fig. 6A but as a function of the chirality number 𝒞. As can be seen in the inset of Fig. 6B, for small shear rates, all curves fall on top of each other. At higher shear rates, only curves with the same α (and hence *G*) fall on top of each other.

This result is important, as it points in this weak shear regime, on the relevant parameters defining the rheotactic drift velocity for a microswimmer. The linear relation between mean transverse rheotactic velocity ${\bar{v}}_{y}$ and shear rate $\dot{\gamma }$, ${\bar{v}}_{y}=l_{0}\dot{\gamma }$, also reveals a length *l*_0_ quantifying how far a bacterium drifts in the vorticity direction at unit shear rate. For our bacterial strain, this length is *l*_0_ ≈ 0.75 μm (see Fig. 2D), and we now know from Eq. 7 and the prefactors shown in the insets of Fig. 6 (B and C) that *l*_0_ is related to the bacteria parameters via $l_{0}\approx 0.1v_{0}\nu /D_{r}^{\text{eff}}$. We note that the prefactor of ∼0.1 is a purely numerical constant that does not depend on the system parameters.

At larger shear rates, the bacteria with lower aspect ratio (α = 3; diamonds) have a higher rheotactic velocity, and the swimmers with higher aspect ratio (α = 10; squares) have a lower rheotactic velocity. This can be understood by looking at higher orders of the stability around the locally marginally stable fixed point around Ψ = π/2. It can be shown that away from the linearly marginable stable fixed point, the attraction toward this point is stronger for smaller α and, hence, the rheotactic velocity is larger.

We also test our scaling for tumbling bacteria and compare the results to nontumbling bacteria in Fig. 6C. We can see that the scaling also works well for tumbling bacteria, where all of the data (black symbols) collapse onto a single curve. The nontumbling and tumbling master curve do not exactly fall on top of each other, which suggests that the simple scaling with $D_{r}^{\text{eff}}$ is not perfect. Note that, as described before, we include tumbling not only as defining an enhanced effective rotational diffusion but also by adding random tumbling events on top of the continuous rotational diffusion. While, in the absence of flow, long-time dynamics may be captured by introducing an effective temperature from stemming from an effective diffusion constant (*23*), this concept fails to be the accurate measure in our situation, where the interplay of flow and tumbling prohibits a simple effective temperature scaling. Again, we obtain a linear regime for small $\dot{\gamma }$ (inset) where the slope is almost the same (but not exactly) as that for the nontumbling bacteria.

Now, we turn to the case of tumbling bacteria in Poiseuille flow by plotting the data extracted from different layers in the channel (and hence local shear rates), as shown in blue in Fig. 6C. The fact that this data collapses together with the simple shear data on a single curve reiterates the fact that, at least for our channel height (*H* = 100 μm), bacterial rheotaxis is determined by the local shear rate.

Last, we show in Fig. 6D how the curves in Fig. 6C approach toward their asymptotic values. As discussed before, bacteria approach the infinite shear limit very slowly, which can be fitted, as a first approximation, to a power law ∼𝒞^−0.5^, as a consequence of the marginally stable fixed point at Ψ = π/2.

### Comparison to experimental and numerical results

Our theoretical analysis allows us now to understand all the observed aspects of the rheotactic velocities in both experiment and Brownian dynamics simulations. First, the aforementioned result that the position of the peaks in *P*(Ψ) for chiral bacteria move closer and closer together with increasing shear rate (Fig. 5D) is clearly observed in the experiments and simulations in Poiseuille flow using tumbling bacteria, as shown for *P*(Ψ) in Figs. 3B and 4B.

Second, we can now understand why the plateau value of the mean rheotactic velocities obtained in experiments and simulations for high shear rates does not approach the mean free swimming speed *v*_0_ (Fig. 2C): The occurrence of a marginally stable fixed point traps bacteria in periodic-like orbits, resulting in broad orientational distributions.

Third, we are able to predict the linear increase of the mean rheotactic velocity for small shear rates, as seen experimentally and in simulations in Fig. 2C, and to determine the dependence of the mean rheotactic velocities on the relevant parameters, resulting in a universal scaling law.

Fourth, we demonstrate that our analysis in simple shear flow qualitatively explains the behavior in Poiseuille flow. We find that the orientation probability distributions *P*(Ψ, Θ) in Poiseuille flow obtained from layers with local shear rate $\dot{\gamma }$ follow the respective distributions in simple shear flow. Tumbling allows bacteria to exploit nonpopular regions in phase space more frequently compared to nontumbling bacteria, but the qualitative results are comparable to the ones of nontumbling bacteria.

## CONCLUSIONS

In this paper, we investigated bacterial rheotaxis in bulk flows using a combined experimental, numerical, and theoretical analysis. Precise microfluidic experiments using *E. coli* bacteria in channel flows provide not only average rheotactic velocities in the vorticity direction as a function of local shear rate in the Poiseuille flow but also accurate velocity and orientation distributions. These results are in perfect agreement with Brownian dynamics simulations and indicate a shift of the peaks of the velocity distributions toward increasing rheotactic velocities with increasing shear rates. These peaks tend toward a maximum rheotactic velocity equal to the bacteria swimming speed and correspondingly to a bacteria orientation perpendicular to the flow direction and thus aligned with the vorticity direction. However, the velocity distributions remain very broad, and average rheotactic velocities always remain significantly below the bacteria swimming speed.

By theoretically analyzing the bacteria orientation dynamics, we elucidate the mechanisms at the origin of the rheotactic behavior and comprehensively show how the interplay between Jeffery orbits, rheotactic torque, and noise leads to the observed rheotactic velocities. Chirality and bacteria geometrical features are encoded into a dimensionless chiral strength that can be used together with a rotational Péclet number to rescale the shear rate. At small shear rate, the resulting chirality number proportionally affects the mean rheotactic drift velocity. Such a regime exists only in the presence of a strong stochastic reorientation process, limiting the natural tendency for the chirality-induced torque to reorient the swimmer in the positive vorticity direction.

When stochasticity is less important, i.e., at higher chirality number or shear rate, the complexity of the dynamical processes will play a central role and limits, in a subtle way, the alignment of the microswimmers perpendicular to the flow. Our theoretical analysis based on a full set of kinematic equations provides a quantitative account for this original dynamical behavior and explains the reorientation dynamics leading to saturation at large shear rates from the marginal stability of a fixed point.

Our work provides a comprehensive understanding of the fundamental physical mechanisms of bacterial rheotaxis. Our analytical model is not specific to *E. coli* bacteria and can, in the future, also be used to describe rheotaxis of other flagellated microorganisms or even a rheotactic torque of a different nature as might exist for artificial microswimmers. It might also be extended to more complex flow environments as soils or porous media and could open interesting perspectives for the design of separation or filtration devices.

## MATERIALS AND METHODS

### Bacterial cultures

We use the wild-type bacteria strain RP437 with fluorescently stained cell bodies, emitting green fluorescence light. These bacteria perform run-and-tumble motion with a typical tumbling frequency of 1 Hz. A rich growth medium (M9G) is used for the bacterial culture and is prepared from 5.64 g of M9 salt, 2 g of glucose, 0.5 g of casamino acid, 50 ml of CaCl2 (1 M), and 1 ml of MgSO4 (1 M) in pure water in a total volume of 500 ml. Culturing with antibiotics (chloramphenicol, 25 μg/ml) is performed in the oven at 30°C at a shaking rate of 200 rpm for about 16 hours until an optical density (OD) of ∼0.5 (measured by Eppendorf D30 at λ = 600 nm) is reached. The uniformity of the bacteria shape and bacteria mobility are verified under the microscope, and subsequently, the bacterial suspension is centrifuged at 5000 rpm for 5 min and redispersed into a motility buffer (0.1 mM EDTA, 1 μM l-methionine, 10 mM sodium lactate, and potassium phosphate buffer with 0.01 M at pH 7) also containing l-serine at a concentration of 0.04 g/ml. Bacteria and motility buffer are density matched by adding Percoll [23% (w/w) colloidal silica particles of 15- to 30-nm diameter in water, coated with polyvinylpyrrolidone] at a 1:1 volume ratio. We work with a very small final density of around 0.15% (v/v) (corresponding to an OD = 1.8).

### Microfluidic devices

Rectangular polydimethylsiloxane (PDMS) channels with the height *H* = 100 μm, width *W* = 600 μm, and length of 20 mm are fabricated using standard soft lithography techniques. A small PDMS layer is spin-coated onto the bottom glass slide to obtain full PDMS channel walls and also to avoid bacteria sticking. A high-precision syringe pump (Cellix ExiGo, Ireland) is used to introduce the bacterial suspension into the microchannel at well-controlled flow rates *Q* = 0, 1, 2, 4, 8, 15, 20, 30, 40, and 50 nl/s, corresponding to wall shear rates of ${\dot{\gamma }}_{\text{wall}}=0,1,2,4,8,15,20,30,40,\text{and}\;50\;s^{-1}$.

### Microscope visualization

The bacterial suspensions are visualized using an inverted microscope (Zeiss Observer, Z1), with an air objective (63×/0.75 LD Plan) and equipped with a Hamamatsu camera (ORCA-Flash 4.0, C11440) at a frame rate of 200 frames per second (fps) at 1024 × 512 pixels (typical field-of-view size of 200 μm by 100 μm). Using a high frame rate is important to track bacteria at high flow rates since bacteria displacements in between two frames need to be small compared to a typical distance between two adjacent bacteria. Because of the use of an air lens, there is a mismatch of refraction index with the solution in the channel and height measurements need to be corrected by a factor of 1.3622. Two methods are used to control the local shear rates in the channel: varying the flow rate *Q* at a given distance from the bottom wall (*z* = 0.1*H* and *z* = 0.2*H*), called *Q* scan, and gradually increasing the distance from the bottom wall with steps δ*z* = 5 × 1.3622 ≈ 6.8 μm at the given flow rates *Q* = 5, 10, and 20 nl/s, called *z* scan. For each position at a given flow rate, 2000 frames are taken as one stack video for the following tracking process. The fluorescent intensity of the RP437 strain is sufficient to allow for a small exposure time of 3 ms.

### Tracking and analysis

Passive polystyrene beads (diameter of 1 μm, emitting red fluorescence light) are mixed with the bacterial suspension at very low concentration (smaller than the bacteria concentration) and are introduced together into the PDMS channel. Bacteria and bead trajectories can now be recorded during the same experiment using either a red or a green filter at a frame rate of 200 fps. Considering the mean speed of bacteria of ∼25 μm/s and a tumbling event about every second, the positions detected are used at time steps of δ*t* = 0.1 s (corresponding to every 20 frames) to determine instantaneous bacteria velocities and orientations.

The depth of field of the used lens has been checked experimentally to be around 2 μm, and observations thus take place within a fluid layer of this thickness. During δ*t* = 0.1 s, bacteria can typically not displace over distances larger than the layer thickness. Hence, we do not filter specific trajectories that are oriented preferentially parallel to the observation plan but capture all trajectories independently of their orientation. The measured trajectories then represent projections of 3D trajectories into the plane of observation.

From the original video stack (2000 frames for each), using the TrackMate routine (Macro in FIJI), the positions of individual bacteria are identified and linked to smooth trajectories. The main parameters used for the tracking routine include Laplacian of Gaussian detector, a blob diameter of 5 pixels, and linear assignment problems (LAP) tracker (with maximum distance varying as a function of the flow rate). After detecting and linking the spots for every bacteria, all the trajectories are saved for the later extraction of the positions [*x^i^*(*t*), *y^i^*(*t*)] from the *i*th trajectory. Similarly, for the passive beads, the positions [*X^i^*(*t*), *Y^i^*(*t*)] are measured, and the mean bead velocity in a specific *z* layer is obtained to be (*V_x_*(*z*), *V_y_*(*z*)) = 〈(*X^i^*(*t* + δ*t*) − *X^i^*(*t*), *Y^i^*(*t* + δ*t*) − *Y^i^*(*t*))/δ*t*〉, where we average over trajectories and time.

For two adjacent position pairs [*x^i^*(*t*), *y^i^*(*t*)], [*x^i^*(*t* + δ*t*), *y^i^*(*t* + δ*t*)] of the *i*th trajectory, the velocity in the *x* direction in a given *z* layer (composed of the swimming and the flow velocity) at time *t* as $v_{x}^{i}(t)=[x^{i}(t+\delta t)-x^{i}(t)]/\delta t$, and the rheotactic velocity $v_{y}^{i}(t)=[y^{i}(t+\delta t)-y^{i}(t)]/\delta t$, is calculated. The unit vector of the bacteria for this *i*th trajectory at the time *t* is defined as $e_{2D}^{i}(t)=({\hat{v}}_{x}^{i}(t),{\hat{v}}_{y}^{i}(t))=((v_{x}^{i}(t)-V_{x}(z))/v_{2D}^{i}(t),v_{y}^{i}(t)/v_{2D}^{i}(t))$ with $v_{2D}^{i}(t)=\sqrt{{\left(v_{x}^{i}-V_{x}(z)\right)}^{2}(t)+{\left(v_{y}^{i}\right)}^{2}(t)}$, where the background flow has been subtracted. The instantaneous orientation angle is then defined as $\Psi^{i}(t)=\text{arctan}\;({\hat{v}}_{y}^{i}(t)/{\hat{v}}_{x}^{i}(t))$.

### Numerical methods

We approximate the shape of a swimming *E. coli* bacterium by an active, “chiral” ellipsoid of 1-μm width and α-μm length, where α is the aspect ratio, and chiral strength ν. In addition to rotational diffusion, tumbling bacteria tumble at exponetially distributed tumble times ∼ exp(−*t*/τ) with τ = 1 s. This is performed by an instantaneous rotation about a random axis around a random angle β drawn from a Gaussian distribution with mean β_0_ = 1.082 rad and SD δβ = 0.454 rad, in accordance with experimentally observed tumbling statistics (*42*).

The equations of motion of a bacterium in flow are given by Eqs. 1 and 2. ℋ is calculated from the translational diffusion tensor $D(\Psi ,\Theta )=\bar{D}1+\Delta DM(\Psi ,\Theta )/2=ℋ\cdot {ℋ}^{T}/2$ via Cholesky decomposition, where M(Ψ,Θ) is a symmetric 3 by 3 matrix [see (*45*)], and $\bar{D}=(D_{1}+D_{2})/2$, Δ*D* = *D*_1_ − *D*_2_, where *D*_1_ = *k_B_Ta*^−1^η^−1^*K*_1_(α) and *D*_2_ = *k_B_Ta*^−1^η^−1^*K*_2_(α) are the respective longitudinal and transversal diffusion coefficients of an ellipsoid of aspect ratio α with shape functions *K*_1_(α) > *K*_2_(α) [see (*12*, *45*)] and with the effective particle (bacterium) radius $a=\sqrt[{3}]{3V_{p}/(4\pi )}$, where *V_p_* is the volume of the particle (bacterium). We use room temperature; hence, *k*_B_*T* = 4.14 pN·nm and buffer viscosity η = 1.28 × 10^−3^ Pa·s. The random numbers ξ*_i_* and $\xi_{i}^{r}$ model Gaussian white noise with zero mean and $\langle \xi_{i}\xi_{j}\rangle =\langle \xi_{i}^{r}\xi_{j}^{r}\rangle =\delta_{\mathrm{ij}}$ (*i* = *x*, *y*, *z*). To compare results with the experiments, we determine the instantaneous velocity of the swimmer at time *t* by using **v**(*t*) = [**r**(*t* + Δ*t*) − **r**(*t*)]/Δ*t*, with Δ*t* = 0.1 s, similar to that in the experiments.

## Supplementary Material

abb2012_SM.pdf

## SUPPLEMENTARY MATERIALS

Supplementary material for this article is available at http://advances.sciencemag.org/cgi/content/full/6/28/eabb2012/DC1
