# Supplementary material for: Chirality-induced bacterial rheotaxis in bulk shear flows
Source: Sci Adv. 2020 Jul 10;6(28):eabb2012. doi: 10.1126/sciadv.abb2012 (PMC7351478; doi:10.1126/sciadv.abb2012)
Supplement: abb2012_SM.pdf [file abb2012_SM.pdf]

Science Advances
AAAS

advances.sciencemag.org/cgi/content/full/6/28/eabb2012/DC1

Supplementary Materials for

Chirality-induced bacterial rheotaxis in bulk shear flows

Guangyin Jing, Andreas Zöttl*, Éric Clément, Anke Lindner

*Corresponding author. Email: andreas.zoettl@tuwien.ac.at

Published 10 July 2020, *Sci. Adv.* **6**, eabb2012 (2020)
DOI: 10.1126/sciadv.abb2012

This PDF file includes:

Supplementary Text
Fig. S1
References

Bacteria swimming speed

The swimming speed of the wild type *E. Coli* strain RP437 (see Methods section) is measured using bacteria tracking (see Methods section) in a microchannel without flow $\sim 20\mu m$ away from the channel walls. 3D velocities are estimated from the 2D projection measurements in the x, y plane, assuming $v_z^2 = \frac{1}{2}(v_x^2 + v_y^2)$. Fig. S1 displays the obtained velocity distribution, with a mean speed of $v_0 \approx 25\mu m/s$. In the simulations a Gaussian velocity distribution with an identical mean velocity is used, as shown by the black dashed line.

Background flow and channel height calibration

As described in the Methods section, passive beads are added to the bacterial suspension to determine the flow profile inside the microchannel of approximate height and width $100\mu m$ and $600\mu m$. We use fluorescent polystyrene beads ($1\mu m$ in diameter emitting red light, Thermo Scientific) at a very low concentration ($1/10$ of the bacteria concentration) to prevent colliding of bacteria and beads. The fact that bacteria and beads fluorescently emit light at different wavelength allows to separately track them during the same experiment.

The first calibration step is to align the $x-$axis with the flow direction, a crucial step to measure precise velocity and orientation distributions. This is simply done by rotating the x and y axis until all flow velocity components along the $y-$ direction become strictly zero.

Second the position of the top and bottom channel walls in z needs to be defined and the z displacement of the stage needs to be linked to the position of the focal plane within the channel height. The latter requires a calibration step, as the use of an air lens induces a mismatch in refraction index with the solution inside the microchannel. For this calibration we use the measured Poiseuille flow profile V_x, first as a function of the stage position, by scanning through the channel height moving the microscope stage in the z direction. By adjusting the symmetric profile we can determine the stage positions that correspond to the top and bottom channel walls to be in focus. This gives an apparent height of the channel of $\bar{H} = 70.5\mu m$. To obtain the correction factor that needs to be applied to obtain the z position inside the microchannel from the z displacement of the stage, the exact channel height needs to be determined. We again use the parabolic flow profile for this purpose. The flow rate imposed by the syringe pump (Cellix ExiGo, Ireland) is known precisely as it has been independently calibrated using a flow sensor. By measuring the maximum flow velocity in the channel center as a function of different applied flow rates we can determine the exact channel height using the Poiseuille flow profile for a rectangular microfluidic channel (46),

$$V_x(z) = 4\frac{3/2\alpha Q}{HW}\frac{z(H-z)}{H^2} \tag{1}$$

with $\alpha = 1/\left[1 - \frac{192}{\pi^5}\left(\frac{H}{W}\right)\sum_{n=1,3,5,...}^{\infty}\frac{1}{n^5}\tanh\left(\frac{n\pi W}{H}\right)\right]$. In this way the real channel height can be determined to be $H = 96.1\mu m$. Note that profilometer measurements lead comparable results

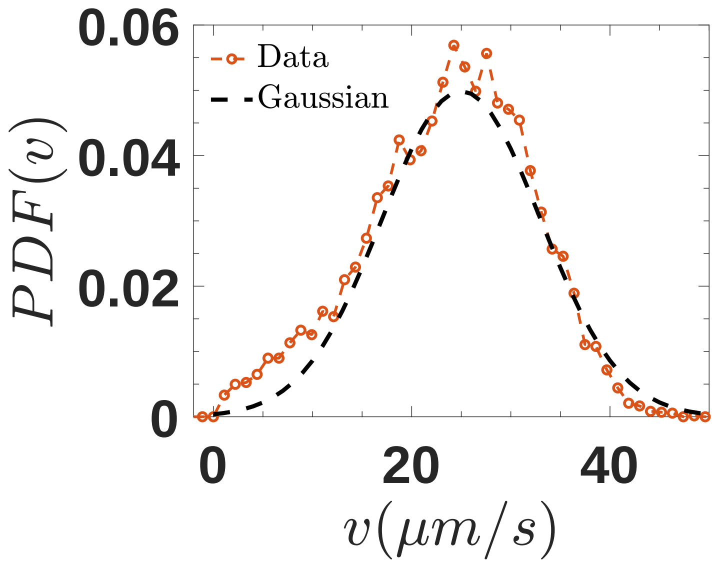

Figure S1: Measured 3D distribution of bacteria swimming speed compared to Gaussian distribution used in simulations (black dashed line).

$(H = 98\mu m)$, but are considered slightly less precise, compared to the adjustment using the flow profile. The conversion factor between the stage displacement and the position within the channel height is thus found to be 1.3622.

REFERENCES AND NOTES

1. J. W. Costerton, P. S. Stewart, E. P. Greenberg, Bacterial biofilms: A common cause of persistent infections. *Science* **284**, 1318–1322 (1999).

2. C. von Eiff, B. Jansen, W. Kohnen, K. Becker, Infections associated with medical devices: Pathogenesis, management and prophylaxis. *Drugs* **65**, 179–214 (2005).

3. R. Bain, R. Cronk, R. Hossain, S. Bonjour, K. Onda, J. Wright, H. Yang, T. Slaymaker, P. Hunter, A. Prüss-Ustün, J. Bartram, Global assessment of exposure to faecal contamination through drinking water based on a systematic review. *Trop. Med. Int. Health* **19**, 917–927 (2014).

4. R. Hatzenpichler, S. A. Connon, D. Goudeau, R. R. Malmstrom, T. Woyke, V. J. Orphan, Visualizing in situ translational activity for identifying and sorting slow-growing archaeal-bacterial consortia. *Proc. Natl. Acad. Sci. U.S.A.* **113**, E4069 (2016).

5. S. Chilukuri, C. H. Collins, P. T. Underhill, Dispersion of flagellated swimming microorganisms in planar Poiseuille flow. *Phys. Fluids* **27**, 031902 (2015).

6. R. Alonso-Matilla, B. Chakrabarti, D. Saintillan, Transport and dispersion of active particles in periodic porous media. *Phys. Rev. Fluids* **4**, 043101 (2019).

7. A. Creppy, E. Clément, C. Douarche, M. V. D'Angelo, H. Auradou, Effect of motility on the transport of bacteria populations through a porous medium. *Phys. Rev. Fluids* **4**, 013102 (2019).

8. F. P. Bretherton, The motion of rigid particles in a shear flow at low Reynolds number. *J. Fluid Mech.* **14**, 284–304 (1962).

9. G. B. Jeffery, The motion of ellipsoidal particles immersed in a viscous fluid. *Proc. R. Soc. Lond. A* **102**, 161–179 (1922).

10. Y. Liu, B. Chakrabarti, D. Saintillan, A. Lindner, O. du Roure, Morphological transitions of elastic filaments in shear flow. *Proc. Natl. Acad. Sci. U.S.A.* **115**, 9438–9443 (2018).

11. J. Einarsson, B. M. Mihiretie, A. Laas, S. Ankardal, J. R. Angilella, D. Hanstorp, B. Mehlig, Tumbling of asymmetric microrods in a microchannel flow. *Phys. Fluids* **28**, 013302 (2016).

12. A. Zöttl, K. E. Klop, A. K. Balin, Y. Gao, J. M. Yeomans, D. G. A. L. Aarts, Dynamics of individual Brownian rods in a microchannel flow. *Soft Matter* **15**, 5810–5814 (2019).

13. G. D’Avino, F. Greco, P. L. Maffettone, Particle migration due to viscoelasticity of the suspending liquid and its relevance in microfluidic devices. *Annu. Rev. Fluid Mech.* **49**, 341–360 (2017).

14. D. Di Carlo, Inertial microfluidics. *Lab Chip* **9**, 3038–3046 (2009).

15. O. du Roure, A. Lindner, E. N. Nazockdast, M. J. Shelley, Dynamics of flexible fibers in viscous flows and fluids. *Annu. Rev. Fluid Mech.* **51**, 539–572 (2019).

16. A. Kumar, M. D. Graham, Margination and segregation in confined flows of blood and other multicomponent suspensions. *Soft Matter* **8**, 10536–10548 (2012).

17. M. Makino, M. Doi, Migration of twisted ribbon-like particles in simple shear flow. *Phys. Fluids* **17**, 103605 (2005).

18. Marcos, H. C. Fu, T. R. Powers, R. Stocker, Separation of microscale chiral objects by shear flow. *Phys. Rev. Lett.* **102**, 158103 (2009).

19. A. Zöttl, H. Stark, Nonlinear dynamics of a microswimmer in Poiseuille flow. *Phys. Rev. Lett.* **108**, 218104 (2012).

20. A. Zöttl, H. Stark, Periodic and quasiperiodic motion of an elongated microswimmer in Poiseuille flow. *Eur. Phys. J. E* **36**, 4 (2013).

21. G. Junot, N. Figueroa-Morales, T. Darnige, A. Lindner, R. Soto, H. Auradou, E. Clément, Swimming bacteria in Poiseuille flow: The quest for active Bretherton-Jeffery trajectories. *Lett. (EPL)* **126**, 44003 (2019).

22. B. Ezhilan, D. Saintillan, Transport of a dilute active suspension in pressure-driven channel flow. *J. Fluid Mech.* **777**, 482–522 (2015).

23. A. Zöttl, H. Stark, Emergent behavior in active colloids. *J. Phys. Condens.Matt.* **28**, 253001 (2016).

24. F. P. Bretherton, L. Rothschild, Rheotaxis of spermatozoa. *Proc. Roy. Soc. B* **153**, 490–502 (1961).

25. V. Kantsler, J. Dunkel, M. Blayney, R. E. Goldstein, Rheotaxis facilitates upstream navigation of mammalian sperm cells. *eLife* **3**, e02403 (2014).

26. C.-K. Tung, F. Ardon, A. Roy, D. L. Koch, S. S. Suarez, M. Wu, Emergence of upstream swimming via a hydrodynamic transition. *Phys. Rev. Lett.* **114**, 108102 (2015).

27. J. Hill, O. Kalkanci, J. McMurry, H. Koser, Hydrodynamic surface interactions enable *Escherichia coli* to seek efficient routes to swim upstream. *Phys. Rev. Lett.* **98**, 068101 (2007).

28. T. Kaya, H. Koser, Direct upstream motility in *Escherichia coli*. *Biophys. J.* **102**, 1514–1523 (2012).

29. N. Figueroa-Morales, G. L. Miño, A. Rivera, R. Caballero, E. Clement, E. Altshuler, A. Lindner, Living on the edge: Transfer and traffic of *E. coli* in a confined flow. *Soft Matter* **11**, 6284–6293 (2015).

30. E. Altshuler, G. Miño, C. Pérez-Penichet, L. del Río, A. Lindner, A. Rousselet, E.Clément, Flow-controlled densification and anomalous dispersion of *E. coli* through a constriction. *Soft Matter* **9**, 1864–1870 (2013).

31. J. Palacci, S. Sacanna, A. Abramian, J. Barral, K. Hanson, A. Y. Grosberg, D. J. Pine, P. M. Chaikin, Artificial rheotaxis. *Sci. Adv.* **1**, e1400214 (2015).

32. L. Ren, D. Zhou, Z. Mao, P. Xu, T. J. Huang, T. E. Mallouk, Rheotaxis of bimetallic micromotors driven by chemical–acoustic hybrid power. *ACS Nano* **11**, 10591–10598 (2017).

33. R. Baker, J. E. Kauffman, A. Laskar, O. E. Shklyaev, M. Potomkin, L. Dominguez-Rubio, H. Shum, Y. Cruz-Rivera, I. S. Aranson, A. C. Balazs, A. Sen, Fight the flow: The role of shear in artificial rheotaxis for individual and collective motion. *Nanoscale* **11**, 10944–10951 (2019).

34. Q. Brosseau, F. B. Usabiaga, E. Lushi, Y. Wu, L. Ristroph, J. Zhang, M. Ward, M. J. Shelley, Relating rheotaxis and hydrodynamic actuation using asymmetric gold-platinum phoretic rods. *Phys. Rev. Lett.* **123**, 178004 (2019).

35. A. Costanzo, R. Di Leonardo, G. Ruocco, L. Angelani, Transport of self-propelling bacteria in micro-channel flow. *J. Phys. Condens. Matter* **24**, 065101 (2012).

36. W. E. Uspal, M. N. Popescu, S. Dietrich, M. Tasinkevych, Rheotaxis of spherical active particles near a planar wall. *Soft Matter* **11**, 6613–6632 (2015).

37. Marcos, H. C. Fu, T. R. Powers, R. Stocker, Bacterial rheotaxis. *Proc. Natl. Acad. Sci. U.S.A.* **109**, 4780–4785 (2012).

38. A. J. T. M. Mathijssen, N. Figueroa-Morales, G. Junot, E. Clément, A. Lindner, A. Zöttl, Oscillatory surface rheotaxis of swimming *E. coli* bacteria. *Nat. Commun.* **10**, 3434 (2019).

39. T. Pedley, J. O. Kessler, Hydrodynamic phenomena in suspensions of swimming microorganisms. *Annu. Rev. Fluid Mech.* **24**, 313–358 (1992).

40. K. Ishimoto, Helicoidal particles and swimmers in a flow at low Reynolds number. *J. Fluid Mech.* **892**, A11 (2020).

41. K. Drescher, J. Dunkel, L. H. Cisneros, S. Ganguly, R. E. Goldstein, Fluid dynamics and noise in bacterial cell–cell and cell–surface scattering. *Proc. Natl. Acad. Sci. U.S.A.* **108**, 10940–10945 (2011).

42. H. C. Berg, D. A. Brown, Chemotaxis in *Escherichia coli* analysed by three-dimensional tracking. *Nature* **239**, 500–504 (1972).

43. R. Rusconi, J. S. Guasto, R. Stocker, Bacterial transport suppressed by fluid shear. *Nat. Phys.* **10**, 212–217 (2014).

44. D. Saintillan, The dilute rheology of swimming suspensions: A simple kinetic model. *Exp. Mech.* **50**, 1275–1281 (2010).

45. D. Matsunaga, F. Meng, A. Zöttl, R. Golestanian, J. M. Yeomans, Focusing and sorting of ellipsoidal magnetic particles in microchannels. *Phys. Rev. Lett.* **119**, 198002 (2017).

46. F. M. White, I. Corfield, *Viscous Fluid Flow* (McGraw-Hill, 2006).
